# A potential prognostic long non-coding RNA signature to predict metastasis-free survival of breast cancer patients

**DOI:** 10.1038/srep16553

**Published:** 2015-11-09

**Authors:** Jie Sun, Xihai Chen, Zhenzhen Wang, Maoni Guo, Hongbo Shi, Xiaojun Wang, Liang Cheng, Meng Zhou

**Affiliations:** 1 College of Bioinformatics Science and Technology, Harbin Medical University, Harbin 150081, PR China; 2 Department of General Surgery, The Fourth Affiliated Hospital, Harbin medical university, Harbin 150081, China

## Abstract

Long non-coding RNAs (lncRNAs) have been implicated in a variety of biological processes, and dysregulated lncRNAs have demonstrated potential roles as biomarkers and therapeutic targets for cancer prognosis and treatment. In this study, by repurposing microarray probes, we analyzed lncRNA expression profiles of 916 breast cancer patients from the Gene Expression Omnibus (GEO). Nine lncRNAs were identified to be significantly associated with metastasis-free survival (MFS) in the training dataset of 254 patients using the Cox proportional hazards regression model. These nine lncRNAs were then combined to form a single prognostic signature for predicting metastatic risk in breast cancer patients that was able to classify patients in the training dataset into high- and low-risk subgroups with significantly different MFSs (median 2.4 years versus 3.0 years, log-rank test p < 0.001). This nine-lncRNA signature was similarly effective for prognosis in a testing dataset and two independent datasets. Further analysis showed that the predictive ability of the signature was independent of clinical variables, including age, ER status, ESR1 status and ERBB2 status. Our results indicated that lncRNA signature could be a useful prognostic marker to predict metastatic risk in breast cancer patients and may improve upon our understanding of the molecular mechanisms underlying breast cancer metastasis.

Breast cancer is the one of the most common malignant tumors in women worldwide^1^. Metastasis to other parts of the body, such as the lung, liver and brain^2^, and breast cancer distant metastasis and recurrence are the primary causes of death for breast cancer patients, with a decrease in long-term survival from 90% to 5%^3^. Currently, more than 80% of patients with metastatic breast cancer receive adjuvant treatment with chemotherapy, which has been shown to increase the 15-year survival rate by 3% to 10% for patients in different age groups^2^,^4^. However, chemotherapy can cause a series of acute and significant side effects that have a major effect on a patient’s life. Therefore, an urgent need exists for the development of prognostic biomarkers able to predict metastatic risk in breast cancer patients which would allow low-risk patients to choose a less aggressive therapy and avoid being over-treated and receiving adjuvant chemotherapy unnecessarily. Some traditional clinicopathological and molecular prognostic markers, such as tumor size, axillary lymph-node status, histological grade, angioinvasion, uPA/PAI1 protein level, steroid-receptor expression and *ERBB2* expression, have already been established and applied in the clinic^2^,^5^, but their predictive ability is only effective in approximately 30% of patients, mainly due to the heterogeneous features of breast cancer at the molecular and genetic levels^6^. More recently, many multi-gene prognostic signatures from microarray gene expression analysis at either mRNA or miRNA levels were shown to predict metastatic risk with greater accuracy than the traditional prognostic criteria^7^,^8^,^9^,^10^,^11^,^12^.

Long non-coding RNAs (lncRNAs), newly discovered members of RNA, are defined as transcripts longer than 200 nucleotides with little or no protein-coding capacity^13^,^14^. Accumulating evidence suggests that lncRNAs are an important layer of the genome regulatory network and play critical roles in a spectrum of biological processes via diverse mechanisms, including chromatin modification, transcriptional regulation and post-transcriptional regulation^15^,^16^. A number of dysregulated lncRNAs have been detected in multiple human cancers^17^,^18^,^19^, and their expression is associated with cancer metastasis and prognosis. For example, the lncRNA *HOTAIR* (*Hox transcript antisense intergenic RNA*) is upregulated in primary breast tumors and metastases, and its overexpression is associated with enhanced breast cancer metastasis^20^. Notably, lncRNA dysregulation was proposed as a hallmark feature in cancer^18^. Recently, several lncRNA signatures were developed as novel predictors of survival in patients with cancer^21^,^22^,^23^,^24^, displaying a similar prognostic power to that of protein-coding RNA and miRNAs, thus providing a new molecular option for cancer diagnosis and prognosis. However, the prognostic power of lncRNA signatures for metastatic risk of breast cancer patients has not yet been investigated.

In this study, we examined the prognostic significance of an lncRNA signature as a predictor of metastatic development in breast cancer patients by repurposing and analyzing the publicly available gene expression profiles of 916 patients from the Gene Expression Omnibus (GEO) database. By using the sample-splitting method and Cox proportional hazards regression model, we identified nine lncRNAs to be significantly associated with patient metastasis-free survival (MFS) in a training dataset. The predictive value of this nine-lncRNA signature was then validated in a testing dataset and two independent datasets. Our results indicated that this nine-lncRNA signature could be a useful prognostic marker to predict metastatic risk in breast cancer patients and may improve our understanding of the molecular mechanisms underlying breast cancer metastasis.

## Results

### Identification of lncRNA genes associated with metastasis in the training dataset

**Figure 1:**
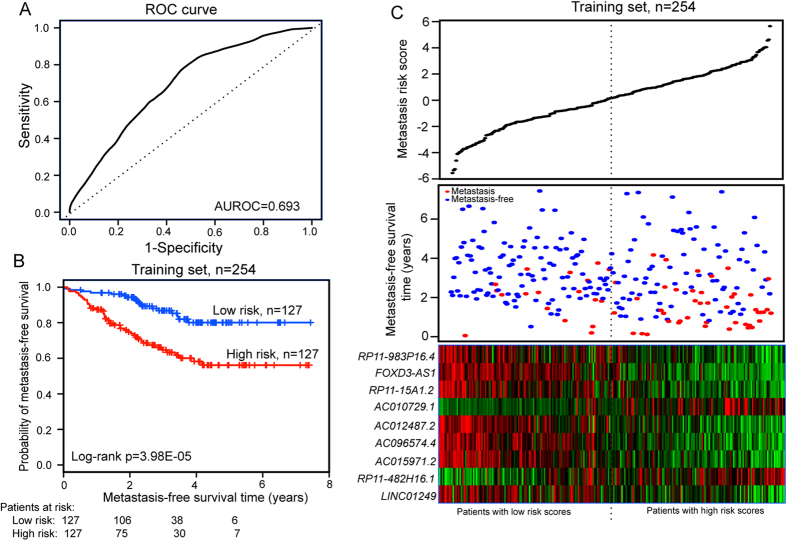
Establishment and performance evaluation of the nine-lncRNA signature for MFS of breast cancer patients in the training dataset. (**A**) The ROC curves for MFS prediction by the nine-lncRNA signature in the training dataset. (**B**) Kaplan-Meier analysis for MFS of breast cancer patients using the nine-lncRNA signature in the training dataset. (**C**) The distribution of the metastasis risk score, patients’ metastasis status and lncRNA expression in the training dataset.

The 508 breast cancer patients from the GSE25066 dataset, which was the largest patient dataset used in this study, were randomly divided into a training dataset (n = 254) and a testing dataset (n = 254). A univariate Cox proportional hazards regression analysis was performed to test whether the expression level of each lncRNA was significantly associated with differences in patient MFS in the training dataset, with MFS as the continuous variable and the expression value of lncRNA as the explanatory variable. Nine lncRNAs (*RP11-482H16.1*, *AC010729.1*, *RP11-983P16.4*, *FOXD3-AS1*, *LINC01249*, *AC096574.4*, *AC015971.2*, *AC012487.2* and *RP11-15A1.2*) were found to be significantly correlated with patient MFS (p < 0.002, Table 1). Of these, *RP11-482H16.1* and *AC010729.1* showed a positive coefficient in univariate analysis, indicating that patients with a higher expression level of *RP11-482H16.1* and *AC010729.1* tended to have a shorter MFS compared with patients with lower expression level of *RP11-482H16.1* and *AC010729.1*. For the seven remaining genes, we observed negative coefficients in univariate analysis, indicating that their high expression was associated with a longer MFS. Furthermore, other clinicopathological and molecular features, such as ER status (p = 0.001; HR = 0.427, 95% CI = 0.257–0.711) and ESR1 status (p = 0.005; HR = 0.486, 95% CI = 0.294–0.803) (Table 2), were also found to be significantly associated with MFS in univariate analysis. Therefore, to further obtain the predictive power of lncRNAs after correcting for these variables, the selected nine lncRNAs and these clinical features were fitted in a multivariate Cox proportional hazards regression model in the training dataset. With the expression levels and regression coefficients of the selected nine lncRNAs, a risk score model was built to predict each patient’s risk of developing metastasis, as follows: metastasis risk score = (0.527 × expression value of *RP11-482H16.1*) + (0.217 × expression value of *AC010729.1*) + (−0.319 × expression value of *RP11-983P16.4*) + (−0.422 × expression value of *FOXD3-AS1*) + (−0.347 × expression value of *LINC01249*) + (−0.355 × expression value of *AC096574.4*) + (−0.390 × expression value of *AC015971.2*) + (−0.389 × expression value of *AC012487.2*) + (−0.549 × expression value of *RP11-15A1.2*). Using receiver operating characteristic (ROC) curve analysis, the prognostic power of the nine-lncRNA signature-based risk score was evaluated against the advent of a metastasis event within 5 years as the defining point in the training dataset of 254 patients. As shown in Fig. 1A, the AUC of the nine-lncRNA risk score applied to the training dataset was 0.693, indicating good performance of the nine-lncRNA signature for predicting metastasis in the training dataset. The 254 patients of the training dataset were then divided into a high-risk group (n = 127) and a low-risk group (n = 127) using the median metastasis risk score as the cut-off. The Kaplan-Meier analysis for MFS as a function of the nine-lncRNA signature showed significant differences in MFS between high-risk and low-risk groups (log-rank test p < 0.001, Fig. 1B). Distribution of the nine-lncRNA risk score, metastasis status and lncRNA expression in patients in the training dataset are shown in Fig. 1C. Median MFS of the high-risk and low-risk groups was 2.4 years and 3.0 years, respectively. At 2 and 6 years, the respective absolute difference in the MFS between the high-risk and low-risk groups was 20.4% (73.9% versus 94.3%) and 24.0% (56.1% versus 80.1%). In univariate analysis, the hazard ratio of the high-risk group versus the low-risk group for MFS was 2.993 (p < 0.001, 95% CI = 1.728–5.184) (Table 2), indicating that the high metastasis risk scores from the nine-lncRNA signature was significantly correlated with shorter MFS.

### Performance assessment of the nine-lncRNA signature for metastasis prediction in the testing dataset, the entire GSE25066 dataset and two independent datasets

**Figure 2:**
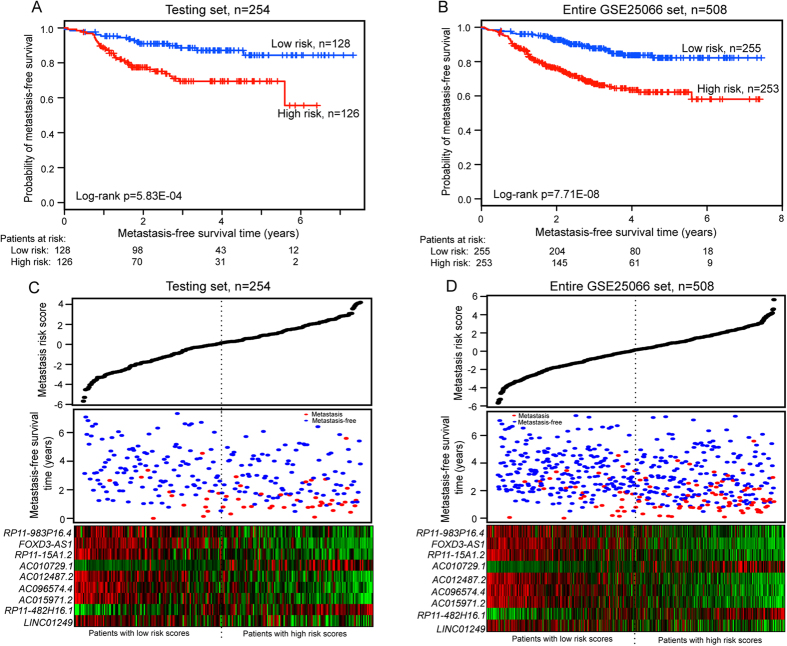
Performance evaluation of the nine-lncRNA signature for MFS of breast cancer patients in the testing dataset and entire GSE25066 dataset. (**A**) Kaplan-Meier curves for patients in the testing dataset (n = 254). (**B**) Kaplan-Meier curves for patients in the entire GSE25066 dataset (n = 508). The two-sided Log-rank test was performed to test the difference for MFS between the high-risk and low-risk groups. The number of patients at risk was listed below the survival curves. (**C**) The distribution of the metastasis risk score, patients’ metastasis status and lncRNA expression in the testing dataset. (**D**) The distribution of the metastasis risk score, patients’ metastasis status and lncRNA expression in the entire GSE25066 dataset.

To validate the prognostic power of the nine-lncRNA signature for the prediction of metastatic risk, the predictive model was applied to the testing dataset (n = 254) and the entire GSE25066 dataset (n = 508). Patients in the testing dataset were divided into a high-risk group (n = 126) and a low-risk group (n = 128) with the nine-lncRNA signature using the same predictive model and threshold from the training dataset. As in the training dataset, MFS of patients in the high-risk group (median 2.6 years) was significantly shorter than that of patients in the low-risk group (median 3.4 years) (log-rank test p < 0.001) (Fig. 2A). MFS of the high-risk group was 77.4% and 55.6% at 2 years and 6 years, respectively, while the corresponding rates in the low-risk group were 90.9% and 84.4%, respectively. The results from univariate analysis of the testing dataset showed that the association of the nine-lncRNA signature with MFS was also significant (HR = 2.794, 95% CI = 1.517–5.148, p < 0.001) (Table 2).

Similar results were obtained from the entire GSE25066 dataset, in which the statistically significant association between MFS and the nine-lncRNA signature risk score was observed by univariate analysis (HR = 2.908, 95% CI = 1.934–4.372, p < 0.001). Patients in the high-risk group (n = 253) had significantly shorter MFS (median 2.3 years) than those in the low-risk group (n = 255) (median 3.1 years) (log-rank test p < 0.001) (Fig. 2B). MFS of the high-risk and low-risk groups was 75.7% and 92.6% at 2 years and 58.1% and 82.2% at 6 years, respectively. The distributions of lncRNA risk score, metastasis status and lncRNA expression of patients in the testing and GSE25066 datasets were then analyzed (Fig. 2C,D). As shown in Fig. 2C,D, the lncRNAs *RP11-482H16.1* and *AC010729.1* were expressed at high levels in patients with high-risk scores, whereas the remaining seven lncRNAs were expressed at high levels in the low-risk patients of the testing and entire GSE25066 datasets.

**Figure 3:**
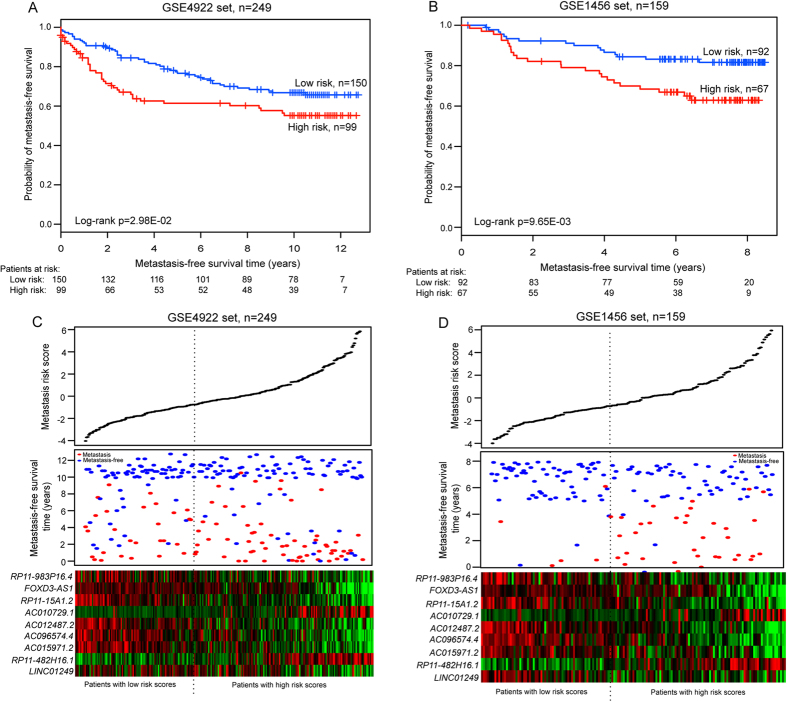
The nine-lncRNA signature-focused risk score in predicting MFS of two independent datasets. Differences in MFS were assessed between high-risk and low-risk groups for the GSE4922 dataset (n = 249) (**A**), and the GSE1456 dataset (n = 159) (**B**). All the p values of Kaplan-Meier analysis were calculated using a two-sided log-rank test. The number of patients at risk was shown below the survival curves. The nine-lncRNA risk score distribution, patients’ metastasis status and heatmap of the nine lncRNA expression profiles were analyzed in the GSE4922 dataset (**C**) and GSE1456 dataset (**D**).

The prognostic value of the nine-lncRNA signature was further validated in two independent breast cancer patient datasets (GSE4922 and GSE1456). The nine-lncRNA signature showed similar results in these datasets, confirming its prognostic power to predict metastatic risk. Using the same predictive model and threshold as in the training dataset, 249 patients of GSE4922 were classified into high-risk (n = 99) and low-risk (n = 150) groups. Patients in the high-risk group exhibited significant shorter MFS (median 7.92 years) than those in the low-risk group (median 10.0 years) (log-rank p = 2.98E-02) (Fig. 3A). The nine-lncRNA risk score also classified patients of the GSE1456 dataset into high-risk (n = 67) and low-risk (n = 92) groups with different MFSs (median 6.3 years versus 7.4 years, log-rank p = 9.65E-03) (Fig. 3B). Univariate analysis was also performed on these two independent datasets. The hazard ratio of the high-risk group versus the low-risk group for MFS was 1.584 (p = 0.031; 95% CI = 1.043–2.404) in the GSE4922 dataset, and 2.257 (p = 0.012; 95% CI = 1.198–4.250) in the GSE1456 dataset. The distribution of lncRNA risk score, metastasis status and lncRNA expression of patients in each independent dataset was consistent with our findings in the training dataset (Fig. 3C,D).

### Independence of metastasis prediction by the nine-lncRNA signature from other clinical variables

**Figure 4:**
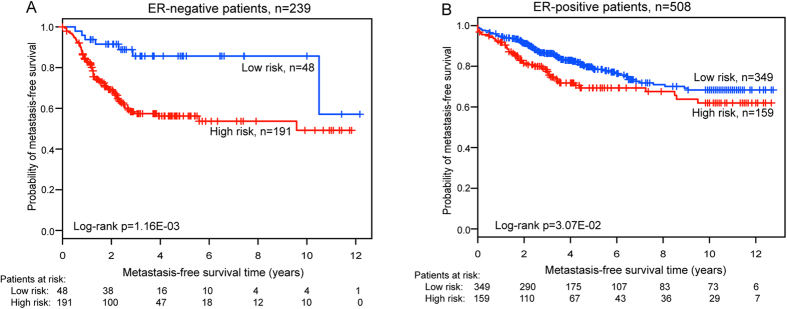
Kaplan-Meier analysis for MFS of breast cancer patients using the nine-lncRNA signature in the subgroups stratified by ER status. (**A**) Kaplan-Meier curves for breast cancer patients with ER-negative status (n = 239). (**B**) Kaplan-Meier curves for breast cancer patients with ER-positive status (n = 508). The differences between the two curves were access by the two-sided log-rank test. The number of patients at risk was listed below the survival curves.

To further investigate whether the predictive ability of the nine-lncRNA signature was independent of other clinical variables, multivariate Cox regression analysis was performed using lncRNA risk score, age, ER status, ESR1 status and ERBB2 status as covariables in the GSE25066 and GSE4922 datasets (no clinical information was available for the GSE1456 patient dataset). The results showed that the nine-lncRNA signature risk score maintained a significant correlation with MFS after adjustment for other clinical variables (HR = 1.791, 95% CI = 1.105–2.904, p = 0.018 for GSE25066; HR = 1.598, 95% CI = 1.018–2.508, p = 0.042 for GSE4922) (Table 3), indicating that the prognostic value of the nine-lncRNA signature was an independent prognostic factor for the prediction of developing metastatic breast cancer. Next, a data stratification analysis was performed according the ER status, which stratified 747 breast patients of three datasets with known ER status into an ER-negative group and an ER-positive group. The risk score of the nine-lncRNA signature further classified 239 patients with ER-negative status into high-risk (n = 191) and low-risk (n = 48) groups with significantly different MFSs (median MFS 2.1 years versus 3.2 years, log-rank test p = 1.16E-03, Fig. 4A). For 508 patients with ER-positive status, the prognostic value of the nine-lncRNA signature between high-risk (n = 159) and low-risk (n = 349) was similar (median MFS time 3.3 years versus 4.0 years, log-rank test p = 3.07E-02, Fig. 4B). These results suggested that the nine-lncRNA signature was also an independent prognostic variable in the subgroups stratified by ER status.

### Functional prediction of the nine-lncRNA signature

**Figure 5:**
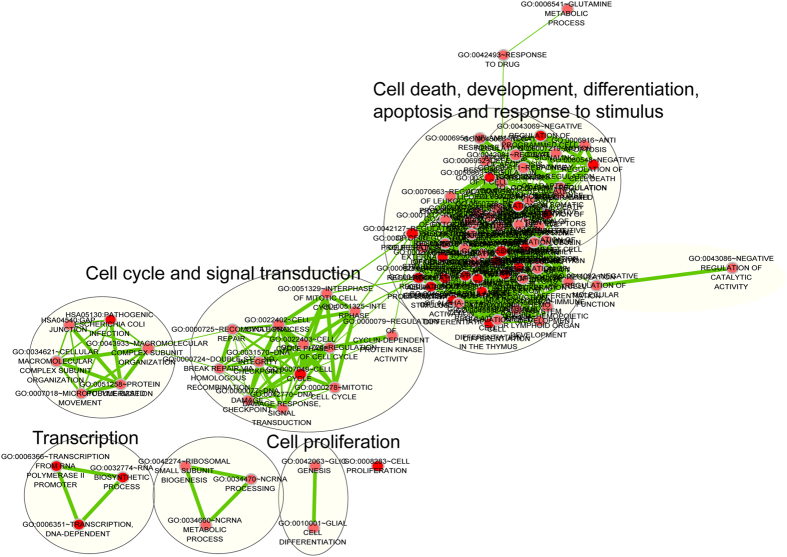
Functional enrichment map of the protein-coding genes co-expressed with prognostic lncRNAs. The enrichment analysis for protein-coding genes positively correlated with prognostic lncRNAs. Each node represents a GO term and an edge represents existing genes shared between connecting GO terms. Node size represents the number of gene in the GO terms. Color intensity is proportional to enrichment significance. The main functional annotations are marked for each cluster of GO terms.

To infer the potential function of the lncRNAs included in the prognostic signature in breast cancer metastasis, an integrative analysis of lncRNA-mRNA functional association was performed, as previously described^24^,^25^,^26^. mRNAs co-expressed with the nine lncRNAs were identified by examining the correlation between expression levels of lncRNAs and those of mRNAs in the 508 breast cancer patients of the GSE25066 dataset. The expression levels of 321 mRNA were positively correlated with those of at least one of the nine prognostic lncRNAs (Pearson correlation coefficient >0.40). The results from Gene Ontology (GO) functional enrichment analysis showed that 321 mRNAs were significantly enriched in 51 GO terms (see Supplementary Table S1 online), which clustered in cell cycle, translation, DNA damage, signal transduction, response to stimulus, cell death, development, differentiation and apoptosis (Fig. 5). Three Kyoto Encyclopedia of Genes and Genomes (KEGG) pathways were found to be enriched by 321 mRNAs, including cell cycle, oocyte meiosis and the p53 signaling pathway (Fig. 5), which are all known to be associated with breast cancer^27^,^28^,^29^.

## Materials and Methods

### Breast cancer patient datasets

Breast cancer patient datasets and corresponding clinical information were collected from the publicly available GEO database. After filtering out patients without metastasis information, a total of 916 patients were selected from three datasets on the Affymetrix HG-U133A platform: 508 patients from GSE25066, 249 patients from GSE4922 and 159 patients from GSE1456. Detailed clinical characteristics of the breast cancer patients in three datasets are summarized in Supplementary Table S2 online.

### Microarray processing and lncRNA expression mining

Raw microarray data files (.CEL files) of the three patient datasets were downloaded from the GEO database, and background adjustment was performed using the Robust Multichip Average (RMA) algorithm by the R package “affy”^30^,^31^. The Affymetrix microarray datasets were normalized by transforming the expression data of each probe into having a mean of 0 and a standard deviation (SD) of 1. lncRNA expression levels from the Affymetrix-based expression profiling of the patients were obtained by repurposing the microarray probes according to a previously described method^32^. First, the probe sets of Affymetrix HG-U133A from the Affymetrix website (http://www.affymetrix.com) were re-mapped to the human genome (GRCh38/hg38) with no mismatch using SeqMap^33^. Second, the chromosomal positions of those probes, which were uniquely mapped to the human genome, were matched to the chromosomal positions of lncRNAs derived from GENCODE (release 21, GRCh38)^34^. A total of 909 probes (or probe sets) and 649 corresponding lncRNA genes were obtained (see Supplementary Table S3 online). Multiple probes (or probe sets) mapping to the same lncRNA were combined by using the arithmetic mean of the values of multiple probes (or probe sets) to form a single lncRNA expression value (log_2_-transformed).

### Statistical analysis

MFS was calculated as the interval between initial primary breast tumor diagnosis and the metastasis event, and patients who died of causes other than breast cancer or were lost to follow-up were censored at that time^35^. To identify lncRNAs predictive of a metastatic event, a univariate Cox proportional hazards regression analysis was performed to evaluate the relationship between the continuous expression level of each lncRNA and patient MFS in the training dataset. Only those lncRNAs correlated with MFS with p-values of <0.002 were considered statistically significant. Multivariate Cox proportional hazards regression was carried out for these selected lncRNAs with MFS as the dependent variable and other clinical information as the covariables. A computational predictive model was built to evaluate each patient’s risk of developing metastasis as follows:


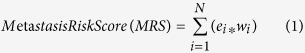


where *N* is the number of prognostic lncRNA genes, *e* is the expression value of the lncRNA, and *w* is the estimated regression coefficient of the lncRNA in the multivariate Cox regression analysis. This predictive model was defined as the linear combination of the expression levels of the selected lncRNAs with their respective Cox regression coefficient as the weight. ROC curves were used to compute the sensitivity and specificity of metastasis prediction of the lncRNA expression-based metastasis risk scores using the R package “survivalROC”. Area under the curve (AUC) values were calculated from the ROC curves. Survival curves were generated using the Kaplan-Meier method, and two-sided log-rank tests were used to assess the differences in MFS between the high-risk and low-risk patient groups using the R package “survival”. Additionally, univariate and multivariate Cox proportional hazards regression, and data stratification analyses were performed to test whether the metastasis risk score was independent of other clinical variables within the available data. The statistical significance was based on p < 0.05 and 95% confidence interval (CI) estimates. All analyses were performed using R/Bio-conductor package.

### Functional prediction of lncRNA signatures

Pearson correlation coefficients were computed between expression levels of lncRNA and those of mRNAs. To reduce the number of false positives, we selected only the top correlated lncRNA/mRNA pairs by setting the correlation threshold to >0.4. The mRNAs co-expressed with lncRNAs were annotated by functional enrichment analyses using the DAVID Bioinformatics Tool (version 6.7)^36^, in which enrichment analysis was carried out using the functional annotation chart and functional annotation clustering options, and was limited to KEGG pathways and GO- FAT biological process (BP) and FAT molecular function (MF) terms. Functional annotation with a p-value of <0.05 and an enrichment score of >2.0 were considered statistically significant. Significant enrichment results were visualized and clustered based on similar function using the Enrichment Map plugin in Cytoscape^37^.

## Discussion

Considerable efforts have been made over the past years to develop a prognostic signature for the prediction of breast cancer metastasis. Variables used for devising such a signature have included traditional clinicopathological and molecular prognostic markers as well as gene expression from DNA-microarray studies. Notably, Van’t veer *et al*.used a supervised classification method to construct a 70-gene signature for risk prediction of distant metastasis through DNA microarray analysis of 117 young breast cancer patients^12^. This 70-gene signature was further validated in a subsequent study of 295 primary breast cancer patients with both lymph-node-negative and lymph-node-positive tumors^11^. Another 76-gene signature, identified from Affymetrix Human U133A GeneChip data of 286 lymph-node-negative patients who had not received adjuvant systemic treatment, was able to predict patients at high risk of distant recurrence^8^. Two biological, knowledge-based metastasis gene signatures [(RKIP pathway metastasis signature (RPMS) and BACH1 pathway metastasis signature (BPMS)] derived from microarray gene expression data, were also reported as prognostic indicators of MFS for triple -negative breast cancer patients^9^,^10^. Recently, using miRNA expression profiling, Marino *et al*.demonstrated that three miRNAs (*miR-183*, *miR-494* and *miR-21*) could be considered markers of metastatic breast cancer risk^7^. However, the molecular and genetic heterogeneity of breast cancer has made the development of a gene signature of breast cancer metastasis to permit personalized adjuvant therapy for individual patients difficult.

A number of studies have demonstrated a critical role of lncRNA deregulation in cancer metastasis, recurrence and prognosis^17^,^19^. Compared with protein-coding genes, lncRNAs exhibit greater tissue-, disease- and developmental stage-specific expression, and their expression is more closely associated with its biological function and tumor status^18^,^38^,^39^,^40^, making lncRNAs attractive emerging molecular biomarkers and therapeutic targets for cancer diagnosis and therapeutics. Indeed, several dysregulated lncRNAs, such as *HOTAIR* and *MALAT-1*, have been found to be associated with breast cancer survival. However, to date, gene expression profile-based prognostic lncRNA signatures for risk prediction of breast cancer metastasis has not been investigated.

In this study, we analyzed and mined the lncRNA expression profiles of 916 breast cancer patients by repurposing the existing microarray expression data on a commonly used microarray platform from the GEO database. We identified nine lncRNAs to be associated with MFS of breast cancer patients in the training dataset by univariate Cox proportional hazards regression analysis and developed a novel predictive model to predict metastatic risk based on the linear combination of expression levels of the nine lncRNAs. The nine-lncRNA signature was first tested in the training dataset by ROC analysis and achieved an AUC of 0.693. Further survival analysis demonstrated a clear separation in the survival curves between patient groups with high-risk or low-risk scores in the training dataset, indicating the predictive power of the signature for breast cancer metastasis. The prognostic value was further validated in the testing dataset and two independent datasets, and significant differences in MFS were observed between high-risk and low-risk groups in the datasets. These results suggest that the prognostic value of the nine-lncRNA signature is in its reproducibility and robustness for predicting breast cancer metastasis risk.

Given the molecular and genetic heterogeneity of breast cancer, we next analyzed whether the prognostic value of the nine-lncRNA signature was independent of clinical characteristics. Cox multivariate analysis for MFS revealed the nine-lncRNA signature to be the significant variable, indicating that the predictive ability of the nine-lncRNA signature for metastatic risk was independent of clinical characteristics, including age, ER status, ESR1 status and ERBB2 status. A recent study also demonstrated that differential expression of lncRNAs between ER-positive and ER-negative subtypes is associated with a different prognosis^25^. The stratified analysis was thus used to assess the ER status-independence of the nine-lncRNA signature in predicting metastatic risk. The results showed that the nine-lncRNA signature exhibited prognostic power for both ER-positive and ER-negative subgroups, in which patients with the same ER status could be classified into high-risk and low-risk groups with significantly different MFS prospects, indicating that the nine-lncRNA signature was an independent prognostic variable in the subgroups stratified by ER status.

A large number of putative lncRNAs have been identified or predicted in humans, though the functions of most lncRNAs remain poorly characterized. To infer the possible functional roles of the nine lncRNAs included in our signature in breast cancer metastasis, we used a computational method integrating lncRNA and mRNA expression profiles. Functional enrichment analysis was performed for 321 mRNAs whose expression positively correlated with the nine lncRNAs at the GO and KEGG pathway levels. We found that predicted function of the nine prognostic lncRNAs belonged to several functional classes, including cell cycle, cell death and apoptosis, as well as cell differentiation and response to stimulus, each of which have been well represented in previously characterized prognostic gene signatures^7^,^8^. Therefore, it is a reasonable inference that the nine lncRNAs may play important functional roles in breast cancer metastasis through interacting with protein-coding genes involved in cell cycle, cell death and apoptosis, cell differentiation, signal transduction and response to stimulus. However, the biological function of these nine lncRNAs in breast cancer metastasis is still not clear and should be investigated in further experimental studies.

In conclusion, our study repurposed and mined large independent breast cancer datasets to reveal a nine-lncRNA signature significantly associated with MFS. With further validation, this lncRNA signature could robustly classify patients with breast cancer into those at high risk of developing metastasis who would benefit from adjuvant therapy and those at low risk of developing metastasis who would be better treated by less aggressive therapeutic treatment. Notably, while this study was in progress, another study performed by Sørensen *et al*.also found that, by the re-annotation of Agilent Human GE 8 × 60K Microarray data, lncRNA profiles can predict metastatic risk in lymph node-negative breast cancer patients^41^. Our results, taken together with Sørensen’s study, signify the potential of lncRNA as novel risk markers for individualized prognosis and treatment decisions for breast cancer patients.

## Additional Information

**How to cite this article**: Sun, J. *et al*.A potential prognostic long non-coding RNA signature to predict metastasis-free survival of breast cancer patients. *Sci. Rep*.**5**, 16553; doi: 10.1038/srep16553 (2015).

## Supplementary Material

Supplementary Information

## Figures and Tables

**Table 1:** lncRNAs significantly associated with the MFS of breast cancer patients in the training set (n = 254).

Gene id	Gene symbol	Chromosome(GRCh38)	P value^a^	Hazard ratio^a^	Cofficient^a^
ENSG00000271894.1	RP11-482H16.1	Chr2:56,147,630–56,386,171(+)	1.40E-03	1.788	0.581
ENSG00000242540.2	AC010729.1	Chr2:5,696,220–5,708,095(+)	1.52E-03	1.373	0.317
ENSG00000257337.4	RP11-983P16.4	Chr12:53,014,596–53,054,438(−)	1.89E-03	0.663	−0.411
ENSG00000230798.3	FOXD3-AS1	Chr1:63,320,884–63,324,441(−)	8.02E-04	0.645	−0.439
ENSG00000231532.3	LINC01249	Chr2:4,628,222–4,656,215(−)	5.77E-04	0.643	−0.441
ENSG00000225057.2	AC096574.4	Chr2:238,231,684–238,255,633(+)	1.09E-03	0.643	−0.441
ENSG00000228363.2	AC015971.2	Chr2:86,562,070–86,618,766(+)	5.82E-04	0.633	−0.456
ENSG00000214184.3	AC012487.2	Chr2:108,507,515–108,534,196(−)	6.76E-04	0.625	−0.470
ENSG00000267191.1	RP11-15A1.2	Chr19:43,902,001–43,926,545(+)	3.40E-04	0.565	−0.570

^a^Derived from the univariable Cox’s proportional-hazards regression analysis in the training set.

**Table 2:** Univariate analysis on the lncRNA signature for MFS.

Variables	HR	95% CI of HR	P value
Training dataset			
lncRNA risk score (low/high)	2.993	1.728–5.184	9.15E-05
Age	1.021	0.996–1.045	0.095
ESR1	0.486	0.294–0.803	0.005
ERBB2	1.509	0.605–3.768	0.378
ER	0.427	0.257–0.711	0.001
Testing dataset			
lncRNA risk score (low/high)	2.794	1.517–5.148	0.001
Age	0.975	0.949–1.002	0.066
ESR1	0.207	0.110–0.392	1.27E–06
ERBB2	1.598	0.633–4.038	0.321
ER	0.271	0.150–0.49	1.59E-05
Entire GSE25066 dataset			
lncRNA risk score (low/high)	2.908	1.934–4.372	2.90E-07
Age	0.998	0.981–1.016	0.860
ESR1	0.336	0.228–0.496	3.79E-08
ERBB2	1.522	0.794–2.916	0.206
ER	0.344	0.234–0.507	6.07E-08
GSE4922 dataset			
lncRNA risk score (low/high)	1.584	1.043–2.404	0.031
Age	0.997	0.982–1.013	0.722
ER	0.858	0.467–1.578	0.623
GSE1456 dataset			
lncRNA risk score (low/high)	2.257	1.198–4.250	0.012

**Table 3:** Multivariate analysis on the lncRNA signature for MFS.

Variables	HR	95% CI of HR	P value
GSE25066 dataset			
lncRNA risk score (low/high)	1.791	1.105–2.904	0.018
Age	1.001	0.983–1.020	0.883
ESR1	0.721	0.387–1.344	0.304
ERBB2	1.981	1.013–3.874	0.046
ER	0.529	0.295–0.951	0.033
GSE4922 dataset			
lncRNA risk score (low/high)	1.598	1.018–2.508	0.042
Age	1.001	0.985–1.017	0.927
ER	1.063	0.561–2.015	0.851
