# Supplementary material for: A potential prognostic long non-coding RNA signature to predict metastasis-free survival of breast cancer patients
Source: Sci Rep. 2015 Nov 9;5:16553. doi: 10.1038/srep16553 (PMC4637883; doi:10.1038/srep16553)
Supplement: Supplementary Information [file srep16553-s1.pdf]

A potential prognostic long non-coding RNA signature to predict metastasis-free survival of breast cancer patients

Jie Sun [1, §], Xihai Chen[2, §], Zhenzhen Wang[1, §], Maoni Guo[1], Hongbo Shi[1], Xiaojun Wang[1], Liang Cheng[1], Meng Zhou [1, *]

[1]College of Bioinformatics Science and Technology, Harbin Medical University, Harbin 150081, PR China

[2]Department of General Surgery，The Fourth Affiliated Hospital, Harbin medical university, Harbin 150081, China

*Corresponding author

Meng Zhou, biofomeng@hotmail.com

College of Bioinformatics Science and Technology, Harbin Medical University, Harbin 150081, PR China

[§]These authors contributed equally to this work

Supplementary Table S1. Functional enrichment analysis of GO and KEGG pathway for mRNA positively correlated with prognostic lncRNAs

Function category	Function term	P value	Fold Enrichment
hsa04110:	Cell cycle	5.08E-03	3.33
hsa04114:	Oocyte meiosis	3.00E-02	2.94
hsa04115:	p53 signaling pathway	4.72E-02	3.4
GO:0031571	G1 DNA damage checkpoint	1.61E-02	15
GO:0005979	regulation of glycogen biosynthetic process	2.58E-02	11.78
GO:0032885	regulation of polysaccharide biosynthetic process	2.58E-02	11.78
GO:0010962	regulation of glucan biosynthetic process	2.58E-02	11.78
GO:0032881	regulation of polysaccharide metabolic process	2.94E-02	11
GO:0021545	cranial nerve development	4.56E-02	8.68
GO:0051184	cofactor transporter activity	2.14E-02	12.97
GO:0004033	aldo-keto reductase activity	2.82E-02	11.24
GO:0031575	G1/S transition checkpoint	3.31E-03	12.94
GO:0014032	neural crest cell development	2.15E-02	6.67
GO:0014033	neural crest cell differentiation	2.15E-02	6.67
GO:0009062	fatty acid catabolic process	2.70E-02	6.11
GO:0007051	spindle organization	4.77E-02	4.89
GO:0007093	mitotic cell cycle checkpoint	7.45E-03	6.39
GO:0000077	DNA damage checkpoint	1.09E-02	5.73
GO:0031570	DNA integrity checkpoint	1.44E-02	5.29
GO:0031329	regulation of cellular catabolic process	2.45E-02	4.51
GO:0009063	cellular amino acid catabolic process	3.47E-02	4.04
GO:0006986	response to unfolded protein	3.97E-02	3.87
GO:0042770	DNA damage response, signal transduction	1.50E-02	4.12
GO:0009894	regulation of catabolic process	3.02E-02	3.44
GO:0051789	response to protein stimulus	4.49E-02	3.08
GO:0001655	urogenital system development	4.96E-02	3
GO:0000075	cell cycle checkpoint	1.30E-03	4.83
GO:0040007	growth	4.90E-02	2.4
GO:0003774	motor activity	1.32E-02	3.17
GO:0046395	carboxylic acid catabolic process	9.17E-04	4.46
GO:0016054	organic acid catabolic process	9.17E-04	4.46
GO:0032269	negative regulation of cellular protein metabolic process	1.69E-02	2.75
GO:0051248	negative regulation of protein metabolic process	2.06E-02	2.65
GO:0006605	protein targeting	4.20E-02	2.3
GO:0050662	coenzyme binding	1.54E-02	2.79
GO:0003924	GTPase activity	3.46E-02	2.4
GO:0009055	electron carrier activity	4.35E-02	2.29
GO:0000226	microtubule cytoskeleton organization	1.43E-03	3.74
GO:0003006	reproductive developmental process	4.93E-02	2.1
GO:0003735	structural constituent of ribosome	3.06E-03	3.35
GO:0006414	translational elongation	1.43E-05	5.99

GO:0051301	cell division	1.89E-02	2.24
GO:0000280	nuclear division	6.78E-04	3.25
GO:0007067	mitosis	6.78E-04	3.25
GO:0048285	organelle fission	9.63E-04	3.12
GO:0000087	M phase of mitotic cell cycle	2.24E-04	3.44
GO:0007017	microtubule-based process	7.19E-04	3.04
GO:0051726	regulation of cell cycle	7.45E-03	2.33
GO:0000279	M phase	9.84E-04	2.67
GO:0006412	translation	1.05E-03	2.66
GO:0000278	mitotic cell cycle	4.20E-04	2.68
GO:0022403	cell cycle phase	1.95E-04	2.66
GO:0022402	cell cycle process	2.47E-04	2.34
GO:0007049	cell cycle	1.66E-04	2.13

Supplementary Table S2. Clinical and pathological characteristics of patients with breast cancer in our study.

Characteristics		Training dataset n=254	Testing dataset n=254	GSE25066 dataset n=508	GSE4922 dataset n=289	GSE1456 dataset n=159
Age, no (%)	(0, 40]	46 (18%)	51 (20%)	97 (19%)	19 (7%)	
	(40, 50]	103 (40%)	77 (30%)	180 (35%)	35 (12%)	
	(50, 60]	58 (23%)	77 (30%)	135 (27%)	52 (18%)	
	(60, 70]	37 (15%)	44 (17%)	81 (16%)	65 (22%)	
	(70, 99)	10 (4%)	5 (2%)	15 (3%)	78 (27%)	
	NA				40 (14%)	
ER, no (%)	P	140 (55%)	157 (62%)	297 (58%)	211 (73%)	
	N	110 (43%)	95 (37%)	205 (40%)	34 (12%)	
	I	2 (1%)	2 (1%)	4 (1%)	0	
	NA	2 (1%)	0	2 (1%)	44 (15%)	
ESR1, no (%)	P	145 (57%)	146 (57%)	291 (57%)		
	N	109 (43%)	108 (43%)	217 (43%)		
ERBB2, no (%)	P	14 (5%)	16 (6%)	29 (6%)		
	N	241 (95%)	238 (94%)	479 (94%)		
Tumor size, no (%)	(0, 3]				211 (73%)	
	(3, 5]				32 (11%)	
	(5, 13]				6 (2%)	
	NA				40 (14%)	
Metastasis event	Yes(1)	62 (24%)	49 (19%)	111 (22%)	89 (31%)	40 (25%)
	NO(0)	192 (76%)	205 (81%)	397 (78%)	160 (55%)	119 (75%)
	NA	0	0	0	40 (14%)	

Supplementary Table S3. The detailed information of probe sets and their corresponding lncRNAs in the Affymetrix HG-U133A platform used in our study

lncRNA ID	Probe
ENSG00000093100.13	212715_s_at
ENSG00000116652.6	215629_s_at
ENSG00000126005.13	213171_s_at
ENSG00000130600.13	217723_x_at
ENSG00000152931.7	"205834_s_at,215972_at"
ENSG00000157306.12	"207699_at,214341_at,218540_at"
ENSG00000173867.8	215919_s_at
ENSG00000175147.9	213752_at
ENSG00000176868.2	212069_s_at
ENSG00000177133.8	207890_s_at
ENSG00000177699.4	215688_at
ENSG00000182109.5	220204_s_at
ENSG00000182873.5	220846_s_at
ENSG00000183562.3	220514_at
ENSG00000186056.7	215053_at
ENSG00000187621.12	219840_s_at
ENSG00000189223.11	"209552_at,213917_at"
ENSG00000197670.5	215861_at
ENSG00000203325.3	214185_at
ENSG00000203386.4	"208725_at,209297_at,209298_s_at,216628_at"
ENSG00000203605.3	206250_x_at
ENSG00000203876.7	222072_at
ENSG00000204929.9	217012_at
ENSG00000204971.3	204437_s_at
ENSG00000205054.4	"207999_s_at,209578_s_at,212421_at,217118_s_at"
ENSG00000205861.9	222277_at
ENSG00000211683.3	221108_at
ENSG00000213904.6	"206576_s_at,206676_at,209498_at"
ENSG00000214184.3	202832_at
ENSG00000215866.5	213739_at
ENSG00000215908.6	221988_at
ENSG00000221819.4	207615_s_at
ENSG00000223442.1	209349_at
ENSG00000223479.3	214276_at
ENSG00000223563.1	219935_at
ENSG00000223647.1	210052_s_at
ENSG00000223653.3	"206837_at,209094_at"
ENSG00000223745.5	201798_s_at
ENSG00000223774.3	211562_s_at
ENSG00000223797.3	206191_at
ENSG00000223799.1	204786_s_at

ENSG00000223814.1	220574_at
ENSG00000223907.1	212758_s_at
ENSG00000223910.1	209538_at
ENSG00000223947.1	"205459_s_at,213462_at,39549_at"
ENSG00000223949.4	"201716_at,213364_s_at,216357_at"
ENSG00000224063.3	"213258_at,214378_at"
ENSG00000224066.1	219585_at
ENSG00000224078.10	"211575_s_at,213128_s_at,213291_s_at,221001_at,221974_at"
ENSG00000224081.5	"208468_at,215103_at,216739_at"
ENSG00000224127.1	212352_s_at
ENSG00000224149.1	212352_s_at
ENSG00000224184.3	216422_at
ENSG00000224209.4	219210_s_at
ENSG00000224445.2	219018_s_at
ENSG00000224459.1	208520_at
ENSG00000224505.2	202815_s_at
ENSG00000224597.7	202565_s_at
ENSG00000224613.4	212424_at
ENSG00000224645.1	202963_at
ENSG00000224660.1	201811_x_at
ENSG00000224854.3	220505_at
ENSG00000224875.2	212931_at
ENSG00000225057.2	205251_at
ENSG00000225087.1	"203620_s_at,208033_s_at,214194_at,220980_s_at"
ENSG00000225206.5	"211953_s_at,211954_s_at"
ENSG00000225313.3	"216811_at,216812_at"
ENSG00000225330.1	206947_at
ENSG00000225420.1	218696_at
ENSG00000225465.7	207934_at
ENSG00000225506.2	"203257_s_at,217319_x_at,217879_at,217880_at"
ENSG00000225623.1	"200659_s_at,207347_at"
ENSG00000225632.1	"221919_at,222040_at"
ENSG00000225667.1	218824_at
ENSG00000225670.3	"208335_s_at,216535_at,217442_at"
ENSG00000225721.3	209408_at
ENSG00000225733.3	217300_at
ENSG00000225762.1	204816_s_at
ENSG00000225889.5	210341_at
ENSG00000226067.4	219022_at
ENSG00000226143.1	213837_at
ENSG00000226245.1	209538_at
ENSG00000226330.1	204484_at
ENSG00000226334.1	215876_at
ENSG00000226419.4	"217570_x_at,220354_at"

ENSG00000226664.1	214324_at
ENSG00000226752.5	215546_at
ENSG00000226785.1	"208370_s_at,215253_s_at,217337_at"
ENSG00000226835.1	218889_at
ENSG00000226883.1	"202345_s_at,207057_at"
ENSG00000226925.1	215561_s_at
ENSG00000226994.5	"201415_at,210365_at,215795_at"
ENSG00000227028.4	"207053_at,217092_x_at"
ENSG00000227082.1	219035_s_at
ENSG00000227110.4	218574_s_at
ENSG00000227117.4	202197_at
ENSG00000227161.1	209308_s_at
ENSG00000227210.1	"206091_at,209684_at"
ENSG00000227218.5	209391_at
ENSG00000227227.1	202351_at
ENSG00000227258.3	"220930_s_at,220931_at"
ENSG00000227292.1	202027_at
ENSG00000227372.8	213340_s_at
ENSG00000227415.1	220090_at
ENSG00000227589.1	209154_at
ENSG00000227617.6	212446_s_at
ENSG00000227907.1	205929_at
ENSG00000227947.1	202738_s_at
ENSG00000227963.1	200071_at
ENSG00000228035.1	206814_at
ENSG00000228192.5	209852_x_at
ENSG00000228237.3	201778_s_at
ENSG00000228242.4	209375_at
ENSG00000228274.3	203450_at
ENSG00000228363.2	202636_at
ENSG00000228389.1	216110_x_at
ENSG00000228395.1	206921_at
ENSG00000228420.1	209045_at
ENSG00000228436.2	"203359_s_at,220605_s_at"
ENSG00000228463.6	"206107_at,206889_at,212504_at,215698_at"
ENSG00000228504.1	218087_s_at
ENSG00000228509.3	209272_at
ENSG00000228613.1	217240_at
ENSG00000228620.1	208359_s_at
ENSG00000228623.3	207068_at
ENSG00000228737.2	216326_s_at
ENSG00000228784.5	210803_at
ENSG00000228794.6	219136_s_at
ENSG00000228839.3	209431_s_at

ENSG00000228843.2 203169_at
ENSG00000228853.1 "208385_at,219027_s_at"
ENSG00000228925.1 212245_at
ENSG00000228971.2 "204557_s_at,209995_s_at,212123_at,219840_s_at"
ENSG00000229160.1 216183_at
ENSG00000229407.3 209353_s_at
ENSG00000229414.2 204487_s_at
ENSG00000229431.1 201376_s_at
ENSG00000229444.1 214392_at
ENSG00000229582.3 220752_at
ENSG00000229621.1 "206064_s_at,209299_x_at"
ENSG00000229846.1
"201221_s_at,209118_s_at,211058_x_at,211072_x_at,213646_x_at,220036_s_at,220774_at"
ENSG00000229953.1 221623_at
ENSG00000229956.7 "205472_s_at,213934_s_at"
ENSG00000230021.5 "202932_at,204142_at,207592_s_at,213645_at,214019_at,217684_at,217690_at"
ENSG00000230023.2 218571_s_at
ENSG00000230027.1 "208753_s_at,208754_s_at"
ENSG00000230084.3 202380_s_at
ENSG00000230148.6 208414_s_at
ENSG00000230185.4 209512_at
ENSG00000230223.4 216391_s_at
ENSG00000230250.1 208521_at
ENSG00000230424.1 215991_s_at
ENSG00000230448.3 "202557_at,202558_s_at,219250_s_at"
ENSG00000230479.1 210689_at
ENSG00000230551.4 "208865_at,213860_x_at"
ENSG00000230615.4 "206175_x_at,207128_s_at,221759_at"
ENSG00000230735.1 205913_at
ENSG00000230798.3 214164_x_at
ENSG00000230817.3 "201759_at,209524_at,209525_at,215055_at,216128_at,216693_x_at"
ENSG00000230864.1 204475_at
ENSG00000230876.4 "201147_s_at,201150_s_at"
ENSG00000231090.1 212386_at
ENSG00000231128.3 "1598_g_at,205115_s_at"
ENSG00000231163.3 220568_at
ENSG00000231204.3 212965_at
ENSG00000231252.1 204566_at
ENSG00000231294.1 206069_s_at
ENSG00000231312.4 "200779_at,204593_s_at,204594_s_at,206947_at,221516_s_at"
ENSG00000231365.3 207593_at
ENSG00000231367.3 "204017_at,207264_at,208359_s_at,208718_at,208719_s_at"
ENSG00000231429.2 219035_s_at
ENSG00000231532.3 216342_x_at

ENSG00000231563.1 220279_at
ENSG00000231607.6 "203659_s_at,205677_s_at"
ENSG00000231609.3 212653_s_at
ENSG00000231671.1 211262_at
ENSG00000231816.1 "202345_s_at,207057_at"
ENSG00000231826.3 216004_s_at
ENSG00000231918.1 "212062_at,219536_s_at"
ENSG00000231949.1 "219781_s_at,221968_s_at"
ENSG00000231964.1 204446_s_at
ENSG00000231987.1 "209120_at,219164_s_at,221773_at"
ENSG00000231992.1 212570_at
ENSG00000231993.1 213579_s_at
ENSG00000231999.4 "212930_at,212978_at,215716_s_at,219707_at"
ENSG00000232021.4 221557_s_at
ENSG00000232284.5 "201088_at,203785_s_at,204428_s_at,210555_s_at,210556_at,211004_s_at,211112_at,212910_at,213141_at,215506_s_at,216476_at,218878_s_at,221111_at"
ENSG00000232298.2 "216388_s_at,220130_x_at"
ENSG00000232377.1 219770_at
ENSG00000232451.1 "202877_s_at,203887_s_at,203888_at"
ENSG00000232633.4 211694_at
ENSG00000232656.5 208881_x_at
ENSG00000232725.1 205142_x_at
ENSG00000232748.3 219047_s_at
ENSG00000232754.1 213579_s_at
ENSG00000232767.1 214434_at
ENSG00000232811.1 220235_s_at
ENSG00000232825.1 "212730_at,214304_x_at"
ENSG00000232828.1 218619_s_at
ENSG00000232878.1 212738_at
ENSG00000232973.9 "216183_at,221178_at"
ENSG00000233005.1 "200676_s_at,200684_s_at,206064_s_at,209299_x_at,212271_at,212965_at"
ENSG00000233070.1 "207246_at,207247_s_at"
ENSG00000233101.8 208414_s_at
ENSG00000233147.1 204574_s_at
ENSG00000233184.4 "217226_s_at,219441_s_at,220974_x_at"
ENSG00000233246.1 210148_at
ENSG00000233290.1 "202856_s_at,208774_at,217685_at"
ENSG00000233359.1 "201439_at,202076_at,216858_x_at,219086_at"
ENSG00000233360.4 213633_at
ENSG00000233399.2 210157_at
ENSG00000233427.1 "209322_s_at,40149_at"
ENSG00000233589.1 214140_at
ENSG00000233695.2 1598_g_at

ENSG00000233706.1	213595_s_at
ENSG00000233718.3	216188_at
ENSG00000233723.5	"202549_at,208601_s_at,217851_s_at,221500_s_at"
ENSG00000233755.1	"220879_at,221001_at"
ENSG00000233818.1	210689_at
ENSG00000233828.3	"215187_at,220828_s_at"
ENSG00000233864.5	214983_at
ENSG00000233891.5	208399_s_at
ENSG00000233896.1	206803_at
ENSG00000233912.1	217852_s_at
ENSG00000233937.4	221897_at
ENSG00000233973.3	220628_s_at
ENSG00000234132.2	217515_s_at
ENSG00000234184.3	"202986_at,216820_at"
ENSG00000234264.1	200605_s_at
ENSG00000234378.1	219811_at
ENSG00000234423.1	204598_at
ENSG00000234425.1	211191_at
ENSG00000234494.5	211736_at
ENSG00000234497.3	"218564_at,218964_at"
ENSG00000234608.5	64432_at
ENSG00000234617.1	209481_at
ENSG00000234684.4	"200709_at,210187_at"
ENSG00000234690.4	"207130_at,209049_s_at"
ENSG00000234807.5	"213429_at,217828_at"
ENSG00000234810.1	"207006_s_at,215715_at,216331_at,217694_at,218381_s_at,219230_at"
ENSG00000234899.7	202935_s_at
ENSG00000234936.1	217346_at
ENSG00000234953.2	217796_s_at
ENSG00000235016.1	201395_at
ENSG00000235106.6	"203245_s_at,203825_at,212547_at,213788_s_at"
ENSG00000235280.2	220354_at
ENSG00000235335.2	220829_s_at
ENSG00000235358.1	211369_at
ENSG00000235373.1	218898_at
ENSG00000235423.6	201938_at
ENSG00000235426.2	212423_at
ENSG00000235437.5	216838_at
ENSG00000235531.7	217590_s_at
ENSG00000235665.3	"201580_s_at,216027_at"
ENSG00000235725.1	217012_at
ENSG00000235848.2	209843_s_at
ENSG00000235872.2	202971_s_at
ENSG00000235910.1	"204156_at,213034_at"

ENSG00000235939.1 207347_at
ENSG00000235954.4 215146_s_at
ENSG00000235958.3 "204598_at,219016_at"
ENSG00000235978.4 216944_s_at
ENSG00000236064.1 209733_at
ENSG00000236065.2 215480_at
ENSG00000236200.3 203205_at
ENSG00000236204.3 "204383_at,210010_s_at,215003_at,216145_at,216285_at,217275_at,217285_at"
ENSG00000236213.1 "209286_at,209287_s_at,209288_s_at,210454_s_at"
ENSG00000236266.1 222088_s_at
ENSG00000236283.1 210432_s_at
ENSG00000236449.1 "202663_at,211940_x_at,213826_s_at,213828_x_at"
ENSG00000236782.3 202533_s_at
ENSG00000236790.3 216027_at
ENSG00000236810.3 213604_at
ENSG00000236856.1 218145_at
ENSG00000236869.1 206314_at
ENSG00000236886.2 "203424_s_at,217424_at,217439_at"
ENSG00000236901.4 221422_s_at
ENSG00000236915.1 216572_at
ENSG00000236948.1 210705_s_at
ENSG00000236963.3 209448_at
ENSG00000237094.9 "216798_at,219664_s_at,221005_s_at"
ENSG00000237188.3 "205776_at,215300_s_at"
ENSG00000237250.3 215970_at
ENSG00000237298.6 208195_at
ENSG00000237301.1 "202386_s_at,208121_s_at,212736_at"
ENSG00000237413.3 206360_s_at
ENSG00000237416.4 216058_s_at
ENSG00000237435.6 206643_at
ENSG00000237491.6 "208954_s_at,218725_at,218993_at,220806_x_at"
ENSG00000237505.4 213063_at
ENSG00000237517.6 215003_at
ENSG00000237667.3 216505_x_at
ENSG00000237781.3 220578_at
ENSG00000237836.3 209439_s_at
ENSG00000237844.1 "216463_at,216618_at"
ENSG00000237938.3 "208543_at,218414_s_at"
ENSG00000237954.5 207436_x_at
ENSG00000238045.7 218300_at
ENSG00000238186.1 219567_s_at
ENSG00000238242.1 219972_s_at
ENSG00000238290.1 "203734_at,209300_s_at,209602_s_at,209603_at,210737_at"
ENSG00000240291.1 "207776_s_at,213714_at"

ENSG00000240553.1	"201715_s_at,221832_s_at"
ENSG00000240618.1	"209092_s_at,212925_at"
ENSG00000240666.1	203434_s_at
ENSG00000241014.1	220608_s_at
ENSG00000241728.3	"203995_at,203996_s_at,214309_s_at"
ENSG00000242349.3	203950_s_at
ENSG00000242540.2	"204913_s_at,204914_s_at,204915_s_at"
ENSG00000242553.1	203635_at
ENSG00000242808.5	214178_s_at
ENSG00000243305.1	"201151_s_at,201152_s_at,201153_s_at"
ENSG00000243415.2	220787_at
ENSG00000243960.1	201421_s_at
ENSG00000244332.1	220085_at
ENSG00000244513.4	213024_at
ENSG00000244558.3	205792_at
ENSG00000245156.1	220964_s_at
ENSG00000245275.5	"207357_s_at,212256_at"
ENSG00000245532.5	214657_s_at
ENSG00000245832.4	208904_s_at
ENSG00000245864.2	"209199_s_at,209200_at"
ENSG00000245928.2	203915_at
ENSG00000246250.2	217869_at
ENSG00000246263.2	208883_at
ENSG00000246316.5	220116_at
ENSG00000246334.2	"202848_s_at,210981_s_at"
ENSG00000246366.4	218701_at
ENSG00000246477.3	204837_at
ENSG00000246777.1	217443_at
ENSG00000246859.2	"201310_s_at,222344_at"
ENSG00000246863.2	215981_at
ENSG00000246889.2	"202066_at,210236_at"
ENSG00000247081.5	218899_s_at
ENSG00000247137.6	220572_at
ENSG00000247993.2	206307_s_at
ENSG00000248049.4	204013_s_at
ENSG00000248138.3	206481_s_at
ENSG00000248525.2	221324_at
ENSG00000249001.3	217067_s_at
ENSG00000249249.1	214658_at
ENSG00000249502.1	217933_s_at
ENSG00000249532.3	215246_at
ENSG00000249717.1	221183_at
ENSG00000249786.5	206073_at
ENSG00000249835.2	204620_s_at

ENSG00000250025.2	208554_at
ENSG00000250135.1	"212101_at,212103_at"
ENSG00000250155.1	202800_at
ENSG00000250186.3	200659_s_at
ENSG00000250608.1	219542_at
ENSG00000250619.1	213169_at
ENSG00000250646.1	203934_at
ENSG00000250733.5	208266_at
ENSG00000250906.1	213419_at
ENSG00000251003.5	219778_at
ENSG00000251018.2	207165_at
ENSG00000251095.4	204466_s_at
ENSG00000251175.3	220063_at
ENSG00000251239.1	207869_s_at
ENSG00000251314.2	205825_at
ENSG00000251448.1	222340_at
ENSG00000251556.1	217810_x_at
ENSG00000251675.1	212812_at
ENSG00000253116.1	220898_at
ENSG00000253352.6	"212337_at,212725_s_at"
ENSG00000253535.3	211239_s_at
ENSG00000253607.1	218172_s_at
ENSG00000253629.1	211685_s_at
ENSG00000253643.3	211239_s_at
ENSG00000253736.1	"201041_s_at,201044_x_at"
ENSG00000253875.1	218125_s_at
ENSG00000253961.1	205667_at
ENSG00000254317.1	217916_s_at
ENSG00000254418.1	"209437_s_at,213993_at,213994_s_at"
ENSG00000254429.1	221861_at
ENSG00000254452.1	220964_s_at
ENSG00000254458.1	219025_at
ENSG00000254488.1	211614_at
ENSG00000254662.1	200957_s_at
ENSG00000254664.1	220571_at
ENSG00000254671.2	216396_s_at
ENSG00000254721.1	202535_at
ENSG00000254873.1	202038_at
ENSG00000254907.1	"201687_s_at,214960_at"
ENSG00000255052.4	222184_at
ENSG00000255125.1	200004_at
ENSG00000255224.1	91684_g_at
ENSG00000255306.1	211004_s_at
ENSG00000255384.1	211775_x_at

ENSG00000255390.1	218892_at
ENSG00000255435.4	"212076_at,212080_at"
ENSG00000255467.1	203531_at
ENSG00000255580.1	220666_at
ENSG00000255644.1	205304_s_at
ENSG00000255723.1	217989_at
ENSG00000255733.3	221111_at
ENSG00000255750.3	204963_at
ENSG00000255794.4	222325_at
ENSG00000255811.1	216579_at
ENSG00000255850.1	204807_at
ENSG00000255929.3	"209892_at,209893_s_at"
ENSG00000255968.1	202662_s_at
ENSG00000256007.1	212516_at
ENSG00000256034.1	201045_s_at
ENSG00000256196.1	201361_at
ENSG00000256234.1	204963_at
ENSG00000256751.3	206312_at
ENSG00000256947.1	202237_at
ENSG00000257000.1	"212375_at,212376_s_at"
ENSG00000257042.1	211756_at
ENSG00000257286.1	221777_at
ENSG00000257337.4	219599_at
ENSG00000257342.1	200714_x_at
ENSG00000257379.1	217794_at
ENSG00000257453.1	218000_s_at
ENSG00000257500.1	207065_at
ENSG00000257520.1	211024_s_at
ENSG00000257545.2	219459_at
ENSG00000257621.5	220701_at
ENSG00000257653.1	209195_s_at
ENSG00000257894.2	"203998_s_at,203999_at"
ENSG00000257913.2	201805_at
ENSG00000258017.1	"211058_x_at,211072_x_at,213646_x_at"
ENSG00000258048.1	201603_at
ENSG00000258092.1	200895_s_at
ENSG00000258199.1	201320_at
ENSG00000258232.2	211750_x_at
ENSG00000258301.3	203940_s_at
ENSG00000258302.2	"212930_at,215716_s_at"
ENSG00000258325.2	200895_s_at
ENSG00000258344.1	"200016_x_at,221919_at,222040_at"
ENSG00000258377.1	211061_s_at
ENSG00000258378.1	218316_at

ENSG00000258384.1 221708_s_at
ENSG00000258407.1 214467_at
ENSG00000258428.3 219757_s_at
ENSG00000258451.1 205141_at
ENSG00000258457.3 219009_at
ENSG00000258520.1 218363_at
ENSG00000258667.1 200989_at
ENSG00000258761.1 210542_s_at
ENSG00000258782.3 "221137_at,221138_s_at"
ENSG00000258851.1 202984_s_at
ENSG00000258871.1 "210270_at,211448_s_at"
ENSG00000258891.1 "202010_s_at,209944_at"
ENSG00000258908.1 201695_s_at
ENSG00000258964.1 200989_at
ENSG00000259006.1 205458_at
ENSG00000259018.1 204400_at
ENSG00000259080.1 220321_s_at
ENSG00000259084.4 219840_s_at
ENSG00000259116.1 213376_at
ENSG00000259138.1 212352_s_at
ENSG00000259172.1 207414_s_at
ENSG00000259248.3 221654_s_at
ENSG00000259291.2 "213657_s_at,37590_g_at,39891_at"
ENSG00000259327.1 204000_at
ENSG00000259357.2 222212_s_at
ENSG00000259370.2 "212701_at,212703_at"
ENSG00000259475.1 "203627_at,214304_x_at"
ENSG00000259627.1 210986_s_at
ENSG00000259661.1 202032_s_at
ENSG00000259673.3 220361_at
ENSG00000259678.1 212820_at
ENSG00000259735.1 215408_at
ENSG00000259736.1 218648_at
ENSG00000259755.1 219441_s_at
ENSG00000259768.5 208033_s_at
ENSG00000259849.1 216726_at
ENSG00000259954.1 "219971_at,221658_s_at"
ENSG00000259976.1 213158_at
ENSG00000259992.1 "209046_s_at,219384_s_at"
ENSG00000260017.1 210057_at
ENSG00000260213.3 222118_at
ENSG00000260252.1 218794_s_at
ENSG00000260269.3 207438_s_at
ENSG00000260372.4 "210066_s_at,210067_at"

ENSG00000260570.1	201806_s_at
ENSG00000260616.4	"213295_at,214272_at,60084_at"
ENSG00000260619.1	216106_at
ENSG00000260661.1	210542_s_at
ENSG00000260743.1	215825_at
ENSG00000260755.1	"204276_at,204277_s_at"
ENSG00000260803.1	217212_s_at
ENSG00000260808.1	217928_s_at
ENSG00000260853.1	217527_s_at
ENSG00000260874.3	"204826_at,204827_s_at"
ENSG00000260886.1	206916_x_at
ENSG00000260917.1	213739_at
ENSG00000260990.1	"206990_at,213841_at"
ENSG00000261002.3	"212202_s_at,212204_at"
ENSG00000261012.2	218492_s_at
ENSG00000261063.1	215301_at
ENSG00000261065.1	219268_at
ENSG00000261067.4	211005_at
ENSG00000261097.1	215738_at
ENSG00000261312.1	213235_at
ENSG00000261351.2	202670_at
ENSG00000261487.1	221864_at
ENSG00000261552.1	211005_at
ENSG00000261582.1	213171_s_at
ENSG00000261771.3	221511_x_at
ENSG00000261790.1	204660_at
ENSG00000262039.1	200659_s_at
ENSG00000262075.3	216608_at
ENSG00000262160.1	207416_s_at
ENSG00000262211.1	"204864_s_at,212195_at"
ENSG00000262668.1	"207338_s_at,217308_at,221402_at"
ENSG00000262769.1	219525_at
ENSG00000262967.1	211717_at
ENSG00000263072.3	206416_at
ENSG00000263218.2	218743_at
ENSG00000263276.1	207416_s_at
ENSG00000263293.2	216261_at
ENSG00000263300.1	219794_at
ENSG00000263585.1	202148_s_at
ENSG00000263711.3	"216703_at,216709_at"
ENSG00000263753.4	215283_at
ENSG00000264339.1	222180_at
ENSG00000264635.1	222180_at
ENSG00000264672.3	210348_at

ENSG00000264860.1	"214597_at,217455_s_at"
ENSG00000265128.1	220774_at
ENSG00000265148.3	218704_at
ENSG00000265263.1	203463_s_at
ENSG00000265408.1	"209667_at,213509_x_at"
ENSG00000265496.3	214291_at
ENSG00000265664.1	221585_at
ENSG00000265678.1	214818_at
ENSG00000265888.1	207324_s_at
ENSG00000266101.1	210185_at
ENSG00000266120.1	202179_at
ENSG00000266283.1	210002_at
ENSG00000266368.1	207959_s_at
ENSG00000266830.1	220654_at
ENSG00000266844.1	207323_s_at
ENSG00000266903.1	212662_at
ENSG00000266929.1	215880_at
ENSG00000267009.4	213836_s_at
ENSG00000267026.3	218885_s_at
ENSG00000267048.1	220715_at
ENSG00000267104.2	221194_s_at
ENSG00000267124.2	"221863_at,44822_s_at"
ENSG00000267165.1	218178_s_at
ENSG00000267169.1	"203488_at,219145_at,47560_at"
ENSG00000267191.1	222028_at
ENSG00000267193.3	208409_at
ENSG00000267219.1	203288_at
ENSG00000267225.1	212880_at
ENSG00000267268.1	215758_x_at
ENSG00000267278.3	205192_at
ENSG00000267280.3	213417_at
ENSG00000267379.1	202910_s_at
ENSG00000267394.1	202692_s_at
ENSG00000267458.1	212952_at
ENSG00000267476.1	208309_s_at
ENSG00000267523.1	218381_s_at
ENSG00000267607.1	207194_s_at
ENSG00000267659.3	217471_at
ENSG00000267698.1	212358_at
ENSG00000267858.3	"204138_s_at,210336_x_at,222097_at,222098_s_at"
ENSG00000267934.1	215344_at
ENSG00000268061.3	208751_at
ENSG00000268087.1	"216423_at,221988_at"
ENSG00000268650.3	206791_s_at

ENSG00000268833.1	"203757_s_at,211657_at"
ENSG00000268983.1	200076_s_at
ENSG00000269072.1	207224_s_at
ENSG00000269082.1	206557_at
ENSG00000269131.1	"212000_at,212001_at,64371_at"
ENSG00000269145.2	213045_at
ENSG00000269176.2	222053_at
ENSG00000269292.1	200623_s_at
ENSG00000269352.1	213690_s_at
ENSG00000269427.1	221988_at
ENSG00000269609.3	213964_x_at
ENSG00000269621.1	209609_s_at
ENSG00000269653.1	210922_at
ENSG00000269707.1	208563_x_at
ENSG00000269749.1	215570_s_at
ENSG00000269793.3	220653_at
ENSG00000269926.1	202887_s_at
ENSG00000269956.1	209467_s_at
ENSG00000269967.1	"208615_s_at,219540_at,220176_at"
ENSG00000269987.1	"212337_at,212725_s_at"
ENSG00000270050.1	204793_at
ENSG00000270240.2	1405_i_at
ENSG00000270792.3	209097_s_at
ENSG00000270820.3	208775_at
ENSG00000271147.5	204793_at
ENSG00000271554.1	221855_at
ENSG00000271763.1	221880_s_at
ENSG00000271894.1	"204092_s_at,221442_at"
ENSG00000271955.1	"204554_at,208399_s_at"
ENSG00000272078.1	220015_at
ENSG00000272106.1	213732_at
ENSG00000272154.2	"216313_at,216355_at,221317_x_at,221410_x_at"
ENSG00000272201.1	213615_at
ENSG00000272438.1	211992_at
ENSG00000272589.1	"214867_at,216345_at"
ENSG00000272758.3	202055_at
ENSG00000272770.1	200813_s_at
ENSG00000273032.1	215003_at
ENSG00000273071.1	"205776_at,215300_s_at"
ENSG00000273112.1	117_at
ENSG00000273192.1	218444_at
ENSG00000273828.1	221528_s_at
ENSG00000274265.2	209700_x_at
ENSG00000274629.1	201370_s_at

ENSG00000275585.1 "205660_at,219022_at"
ENSG00000276255.2 221109_at
ENSG00000276980.1 221159_at
ENSG00000277688.1 213637_at
ENSG00000277801.1 218031_s_at
ENSG00000278921.1 220364_at
ENSG00000279010.1 209843_s_at
ENSG00000279027.1 220569_at
ENSG00000279033.1 218152_at
ENSG00000279038.1 "216847_at,216848_at"
ENSG00000279117.1 221861_at
ENSG00000279154.1 216732_at
ENSG00000279159.1 202197_at
ENSG00000279160.1 205975_s_at
ENSG00000279166.1 215713_at
ENSG00000279324.1 220871_at
ENSG00000279328.1 220824_at
ENSG00000279338.1 "201147_s_at,201150_s_at"
ENSG00000279364.1 220608_s_at
ENSG00000279428.1 203268_s_at
ENSG00000279516.1 211455_at
ENSG00000279636.1 220701_at
ENSG00000279689.1 202361_at
ENSG00000279726.1 "210572_at,211365_s_at,211870_s_at"
ENSG00000279821.1 215534_at
ENSG00000279861.1 208751_at
ENSG00000279863.1 220670_at
ENSG00000279977.1 216423_at
ENSG00000279978.1 206839_at
ENSG00000279980.1 "211457_at,211458_s_at"
ENSG00000280023.1 207779_at
ENSG00000280029.1 "208504_x_at,216313_at,216355_at,221317_x_at,221408_x_at,221450_x_at"
ENSG00000280061.1 206403_at
ENSG00000280069.1 222214_at
ENSG00000280120.1 215731_s_at
ENSG00000280143.1 203118_at
ENSG00000280211.1 "206845_s_at,217642_at"
ENSG00000280397.1 210703_at
ENSG00000280416.1 209667_at
